# Prognostic significance of the methylation of Wnt pathway antagonists—*CXXC4*, *DACT2*, and the inhibitors of sonic hedgehog signaling—*ZIC1*, *ZIC4*, and *HHIP* in head and neck squamous cell carcinomas

**DOI:** 10.1007/s00784-016-1946-5

**Published:** 2016-08-23

**Authors:** Jarosław Paluszczak, Dorota Wiśniewska, Magdalena Kostrzewska-Poczekaj, Katarzyna Kiwerska, Reidar Grénman, Daniela Mielcarek-Kuchta, Małgorzata Jarmuż-Szymczak

**Affiliations:** 1 Department of Pharmaceutical Biochemistry, Poznan University of Medical Sciences, ul. Święcickiego 4, 60-781 Poznań, Poland; 2 Department of Cancer Genetics, Institute of Human Genetics, Polish Academy of Sciences, Poznań, Poland; 3 Department of Otorhinolaryngology, Head and Neck Surgery and Department of Medical Biochemistry, Turku University Central Hospital and Turku University, Turku, Finland; 4 Department of Otolaryngology and Laryngological Oncology, Poznan University of Medical Sciences, Poznań, Poland

**Keywords:** Head and neck cancer, DNA methylation, Shh pathway, Wnt pathway, *ZIC4*

## Abstract

**Objectives:**

Aberrations in Wnt and Shh signaling pathways are related to the pathogenesis of head and neck carcinomas, and their activation frequently results from epigenetic alterations. This study aimed to assess the frequency of methylation of negative regulators of Wnt signaling: *CXXC4*, *DACT2*, *HDPR1*, and *FBXW11* and Shh signaling: *HHIP*, *PTCH1*, *SUFU*, *ZIC1*, and *ZIC4* and correlate it with clinicopathological features in this group of patients.

**Materials and methods:**

Methylation-specific PCR was used to detect gene promoter methylation, and real-time PCR was used to assess gene expression level.

**Results:**

The analysis of the occurrence of gene promoter methylation in head and neck carcinoma cell lines indicated that *CXXC4*, *DACT2*, *HHIP*, *ZIC1*, and *ZIC4* are methylated in these tumors. These genes were further analyzed in tumor sections from oral and laryngeal cancer patients. Gene methylation rate was higher in laryngeal tumors. The methylation index in tumor samples correlated with the overall survival in a subgroup of oral cancer patients who died of the disease. Moreover, *ZIC4* methylation correlated with lymph node involvement in oral cancer patients.

**Conclusions:**

Our findings corroborate that the activation of Wnt signaling in head and neck squamous cell carcinoma (HNSCC) is related to epigenetic silencing of its negative regulators. Moreover, the results indicate that the same mechanism of activation may operate in the case of Shh signaling.

**Clinical relevance:**

The methylation of *ZIC4* may be considered a new prognostic marker in oral cavity and oropharyngeal tumors. Further investigations should determine the diagnostic significance of methylation of *ZIC4*, *HHIP*, and *DACT2* in head and neck carcinomas.

## Introduction

Head and neck cancers consist approximately 6 % of all cancer cases in Poland with nearly six thousand new cases annually [1, 2]. Despite recent advancements in oncology, the five-year survival rates in head and neck cancers have remained unchanged and are estimated overall around 50–60 %. The prognosis is much worse in the case of patients suffering from more advanced (regional and distant) tumors. New therapeutic strategies and diagnostic markers are necessary for the improvement of treatment outcomes. The molecular biology of head and neck squamous cell carcinomas (HNSCC) which are the most prevalent type of head and neck malignancy is increasingly well understood what currently forms the basis for the search of both new biomarkers and novel chemotherapeutic strategies. Among the many molecular aberrations observed in HNSCC, changes in the activity of signaling pathways such as TGFβ, Ras/PI3K/AKT/mTOR, Notch, or NFκB which are responsible for the regulation of cell proliferation, migration, and cell death emerge as important factors associated with both the pathogenesis and progression of the disease [3–5]. Recently, aberrations in the function of sonic hedgehog (Shh) and canonical Wnt pathways have been also implicated in the development of HNSCC.

Canonical Wnt signaling regulates the nuclear translocation of β-catenin. In unstimulated cells, β-catenin is sequestered in the cytoplasm by an inhibitory complex which consists of axin, APC, casein kinase and glycogen synthase kinase—GSK3β. In these conditions, β-catenin undergoes phosphorylation and is targeted for proteasomal degradation. The binding of Wnt ligands to membrane frizzled/LRP receptors causes the destabilization of the inhibitory complex via activation of disheveled and leads to the release of β-catenin which enters the nucleus and binds to TCF/LEF transcription factors. This stimulates the transcription of various genes responsible for cell cycle regulation and cell migration including *CCND1* (encoding cyclin D1)*, survivin, MMP-7* (encoding matrix metalloproteinase 7), and *c-Myc* [6]. The precise regulation of the pathway is also secured by the activity of a group of proteins which antagonize the progression of the pathway in a stage-specific manner. The activation of canonical Wnt pathway is detected already in precancerous lesions [7, 8], and its dysregulation may increase with tumor progression attributing to enhanced cancer cell migration and thus worse prognosis [9–11]. The activation of Wnt signaling in HNSCC is rarely the result of genetic mutations but usually occurs due to the hypermethylation of genes encoding the negative regulators of the pathway [12–15]. The methylation of Wnt pathway antagonists such as *SFRP1–5*, *DKK1–3*, *WIF1*, *DACH1*, and *PPP2R2B* has been frequently observed, and it was associated with worse prognosis [15–18]. The biological importance of Wnt dysregulation for the development of HNSCC is corroborated by the fact that the growth of tongue cancer cells is inhibited by the silencing of *CTNNB1* gene which encodes β-catenin [19].

Sonic hedgehog is another signaling pathway active in embryonal and somatic stem cells which undergoes activation in human cancers [20] and may have crosstalk with Wnt signaling. The binding of Shh ligand to patched (*PTCH1*) protein relieves the inhibition of smoothened (SMO) which is then able to relieve the sequestration of a Gli1 transcription factor by a cytoplasmic complex which includes SUFU (suppressor of fused homolog). This subsequently induces Gli1 translocation to the nucleus where it stimulates the expression of genes related to cell proliferation and cell cycle regulation. Evidence for the implication of Shh pathway dysregulation in the pathogenesis of HNSCC has only recently started to accumulate [21–24]. Importantly, Shh activation was associated with disease progression and worse prognosis in oral squamous cell carcinoma (OSCC) [25]. Based on these findings, Shh signaling has been indicated as a new therapeutic target in this tumor type [26]. Indeed, treatment of HNSCC cells with Shh inhibitor cyclopamine suppressed cell growth and enhanced the cytotoxic effects of cisplatin and docetaxel [27]. However, the molecular mechanisms leading to the activation of Shh in HNSCC have not been well studied although it is suggested that this may occur due to epigenetic changes [28].

The association between the dysregulation of Wnt and Shh pathways and the pathogenesis of HNSCC implicates the possibility of therapeutic targeting HNSCC by modulating the activity of these pathways. This however requires a thorough understanding of the molecular mechanisms which lead to pathway activation, and epigenetic mechanisms have emerged as the possibly most important factor which attributes to this phenomenon. Relatively, much is known about the effect of epigenetic silencing of several negative regulators of Wnt pathway on its activation in HNSCC although the studies have focused only on few regulatory genes so far. On the other hand, the contribution of epigenetic mechanisms to Shh activation in HNSCC has not been well studied and requires exploration. Most importantly, epigenetic silencing of negative regulators of Wnt and Shh pathways may have diagnostic significance. Thus, the aim of the present study was to assess the frequency of methylation of the selected negative regulators of Wnt signaling: *CXXC4*, *DACT2*, *HDPR1* (*DACT1*), and *FBXW11* and Shh signaling: *HHIP*, *PTCH1*, *SUFU*, *ZIC1*, and *ZIC4* which were either rarely or never under study in head and neck cancers previously and correlate it with clinicopathological features in a group of HNSCC patients.

## Materials and methods

### Cell lines

Thirty-six cell lines which were derived from squamous cell carcinomas of the head and neck region at the University of Turku, Finland [29] were analyzed. The characteristics of the patients whose surgically removed tumor sections were used for the establishment of the respective cell lines are presented in Table 1.

**Table 1:** Characteristics of the patients and the derived cell lines

Name of cell line	Sex	Age	Primary tumor location	TNM staging	Specimen site	Type of lesion	Histological grade
UT-SCC-4	F	43	Hypopharynx/supraglottic larynx	T_3_N_0_M_0_	Neck	met	G2
UT-SCC-6A	F	51	Supraglottic larynx	T_2_N_1_M_0_	Larynx	rec	G1
UT-SCC-6B	F	51	Supraglottic larynx	T_2_N_1_M_0_	Neck	met	G1
UT-SCC-7	M	67	Skin (temporal region)	T_1_N_0_M_0_	Neck	met	G2
UT-SCC-8	M	42	Supraglottic larynx	T_2_N_0_M_0_	Larynx	pri	G1
UT-SCC-10	M	62	Tongue scc	T_1_N_0_M_0_	Tongue	pri	G2
UT-SCC-16A	F	77	Tongue scc	T_3_N_0_M_0_	Tongue	pri	G3
UT-SCC-16B	F	77	Tongue scc	T_3_N_0_M_0_	Neck	met	G3
UT-SCC-17	M	65	Supraglottic larynx	T_2_N_0_M_0_	Sternum	met	G3
UT-SCC-19A	M	44	Glottic larynx	T_4_N_0_M_0_	Larynx	pri	G2
UT-SCC-19B	M	44	Glottic larynx	T_4_N_0_M_0_	Larynx	pri (per)	G2
UT-SCC-20A	F	58	Floor of mouth	T_1_N_0_M_0_	Floor of mouth	pri (per)	G2
UT-SCC-20B	F	58	Floor of mouth		Floor of mouth	resid	G2
UT-SCC-22	M	79	Glottic larynx	T_1_N_0_M_0_	Larynx	rec	G2
UT-SCC-23	M	66	Transglottic larynx	T_3_N_0_M_0_	Larynx	pri (per)	G1
UT-SCC-24A	M	41	Tongue scc	T_2_N_0_M_0_	Tongue	pri	G2
UT-SCC-24B	M	41	Tongue scc		Neck	met (per)	G2
UT-SCC-29	M	82	Glottic larynx	T_2_N_0_M_0_	Larynx	pri	G1
UT-SCC-34	M	63	Supraglottic larynx	T_4_N_0_M_0_	Supraglottic larynx	pri	G1
UT-SCC-35	M	50	Glottic larynx	T_2_N_0_M_0_	Larynx	resid	G2
UT-SCC-36	M	46	Floor of mouth	T_4_N_1_M_0_	Floor of mouth	pri	G3
UT-SCC-38	M	66	Glottic larynx	T_2_N_0_M_0_	Larynx	pri	G2
UT-SCC-45	M	76	Floor of mouth	T_3_N_1_M_0_	Floor of mouth	pri	G3
UT-SCC-47	M	78	Floor of mouth	T_2_N_0_M_0_	Floor of mouth	pri	G3
UT-SCC-56	M	62	Floor of mouth	T_x_N_2_M_0_	Floor of mouth	rec	G2–G3
UT-SCC-58	M	63	Transglottic larynx	T_4_N_1_M_0_	Neck	met (skin)	G1
UT-SCC-85	M	55	Tongue scc	T_3_N_0_M_0_	Tongue floor of mouth	rec	G2
UT-SCC-90	M	35	Tongue scc	T_1_N_0_M_0_	Floor of mouth	rec/met	G2
UT-SCC-100	M	70	Gingival scc	rT_3_	Mucosae bucchae	rec	G3
UT-SCC-103	M	51	Glottic larynx	T_3_N_0_M_0_	Larynx	pri (per)	G2
UT-SCC-104	M	80	Supraglottic larynx	T1N_2A_M_0_	Neck	met	G2
UT-SCC-106A	M	59	Glottic larynx	T_1A_N_0_M_0_	Larynx	pri	G2
UT-SCC-106B	M	59	Glottic larynx	rT_3_N_0_M_0_	Larynx	rec	G3
UT-SCC-107	M	46	Supraglottic larynx	T_4_N_2C_M_0_	Larynx	pri	G2
UT-SCC-108	M	68	Supraglottic larynx	T_2_N_0_M_0_	Larynx	pri	G3
UT-SCC-116	M	60	Supraglottic larynx	T_4_N_1_M_0_	Larynx	pri	G2

*F* female, *M* male, *scc* squamous cell carcinoma, *pri* primary, *per* persistent, *rec* recurrent, *met* metastasis, *resid* residual

### Patients

The study group consisted of 42 patients with primary oral/oropharyngeal squamous cell carcinoma (localized mostly in tongue or tonsils) and 30 patients with primary laryngeal squamous cell carcinoma (LSCC) who were primarily treated surgically at the Department of Otolaryngology and Laryngological Oncology, Poznan University of Medical Sciences. In the case of oral cancer patients, a tumor section along with a fragment of macroscopically normal surgical margin was obtained from each patient for analysis. Only tumor sections which contained at least 80 % of cancer cells were qualified for further investigation. Patient follow-up observation usually covered at least 3 years post-operation. Eighteen OSCC and 12 LSCC patients died of the disease during follow-up. The study was approved by the local ethics committee at Poznan University of Medical Sciences. The clinical characteristics of the study group are presented in Table 2.

**Table 2:** Clinical characteristics of patients in the study group

		OSCC	LSCC
Age	Range	28–84	39–79
	Mean	57	57
Sex	Female	4	2
	Male	38	28
TNM staging	T1	4	0
	T2	23	2
	T3	8	17
	T4	7	11
	N0	20	21
	N1	13	4
	N2	7	5
	N3	2	0
	M0	42	30
G histological stage	G1	9	8
	G2	26	21
	G3	7	1
Overall survival time (months)	Range	0–72	4–93
	Mean	30	54
	Tumor-related death	18	12
Other	Recurrence	6	11
	Metastasis	1	1
	Second primary tumor	2	0

### Methylation-specific PCR

DNA samples from the early passage cell lines and from a clinical material were prepared using standard phenol/chloroform extraction protocol. Methylation-specific PCR (MSP) was used for the analysis of gene promoter methylation [30]. Prior to amplification, all the DNA samples underwent bisulfite conversion using an EZ DNA methylation Kit (ZymoResearch, USA) according to manufacturer’s protocol. Primer sequences for the analysis of *CXXC4*, *FBXW11*, *SUFU*, *ZIC1*, and *ZIC4* were designed using the MethPrimer online software [31] and are presented in Table 3. Primer sequences used for the analysis of *DACT2*, *HDPR1*, *HHIP*, and *PTCH1* were previously described [32–35]. All the primers were obtained from the oligonucleotide synthesis facility at the Institute of Biochemistry and Biophysics, Polish Academy of Sciences. DNA derived from white blood cells of healthy donors and fully methylated human genomic DNA (Thermo Scientific, USA) were used as negative and positive MSP controls, respectively. Moreover, samples of genomic DNA extracted from early passage primary culture of human oral keratinocytes and human tracheal epithelial cells (ScienCell Research Laboratories, USA) were used as normal controls for comparison. PCR reactions were performed in a T100 thermal cycler (Bio-Rad Laboratories, USA) using HOT FIREPol Polymerase (Solis BioDyne, Estonia). The amplification protocol was as follows: initial enzyme activation for 15 min at 95 °C followed by 40 cycles of 95 °C for 30 s, *X*°C for 30 s and 72 °C for 30 s, and the final elongation at 72 °C for 5 min, where *X* stands for the appropriate annealing temperature as described in Table 3. PCR products were resolved on 2 % agarose gels in the presence of the Midori Green DNA stain (Nippon Genetics, Japan) and subsequently visualized under UV light illumination. The resulting electrophoregrams were interpreted as previously [17]. In the case of the cell lines, the presence of the band only in the reaction with the starters specific for the unmethylated sequence was treated as lack of gene methylation (U), whereas the presence of the band only in the reaction with the starters specific for the methylated sequence was treated as full gene methylation (M). Partial methylation (M/U) was detected when bands were observed in both reactions. Because DNA samples extracted from tumor sections cannot be treated as derived from a rather homogenous population of cancer cells (as in the case of cell lines), thus a simplified interpretation was used. Gene methylation (M) was detected in each case when the band in the reaction with the starters specific for the methylated sequence was present. Lack of gene methylation (U) was detected in each case when the band in the reaction with the starters specific for the methylated sequence was missing and only the band in the reaction with the starters specific for the unmethylated sequence was present.

**Table 3:** Sequences of the starters used in methylation-specific PCR designed using MethPrimer

Gene	Sequence		Ta
*CXXC4*	MF	5′ GGAGAGAGAGAGGAGAGATTTTTC 3′	60 **°**C
	MR	5′ ATAAAAACGCGAATCTATTATCGAT 3′	
	UF	5′ AGAGAGAGAGGGAGAGATTTTTTGG 3′	56 **°**C
	UR	5′ ATAAAAACACAAATCTATTATCAAT 3′	
*FBXW11*	MF	5′ TAGGAAGTTAAGAGAAGTTAGTCGG 3′	59 **°**C
	MR	5′ CACTAAACGATAAACGTAAAACGTA 3′	
	UF	5′ TAGGAAGTTAAGAGAAGTTAGTTGG 3′	59 **°**C
	UR	5′ CACTAAACAATAAACATAAAACATA 3′	
*SUFU*	MF	5′ GTTTCGGGGAGTTTTATTTATC 3′	*60 °C*
	MR	5′ GAAAACCGAAAAAACAATCG 3′	
	UF	5′ GTTTTGGGGAGTTTTATTTATTGA 3′	*60 °C*
	UR	5′ AAACAAAAACCAAAAAAACAATCA 3′	
*ZIC1*	MF	5′ TTTCGTTAAACGTTTATTCGTTTC 3′	62 **°**C
	MR	5′ CACCTAACCTCCTAAAAACCTACG 3′	
	UF	5′ TTTGTTAAATGTTTATTTGATTTTGT 3′	54 **°**C
	UR	5′ TCACCTAACCTCCTAAAAACCTACA 3′	
*ZIC4*	MF	5′ CGGTTCGGTTAGGAAATTTATC 3′	62 **°**C
	MR	5′ AACCAAAAAAACGAAAAACGAC 3′	
	UF	5′ AGGTGGTTTGGTTAGGAAATTTATT 3′	60 **°**C
	UR	5′ AAAAACCAAAAAAACAAAAAACAAC 3′	

*M* methylated, *U* unmethylated, *F* forward, *R* reverse, *Ta* annealing temperature

### Quantitative PCR

Standard phenol/guanidine thiocyanate extraction protocol served for the preparation of total RNA samples from early passage cell lines. An RNA sample isolated from first passage primary oral keratinocytes (ScienCell Research Laboratories, USA) was used as a calibrator in gene expression quantification. All RNA samples were reverse transcribed using the RevertAid First Strand cDNA Synthesis Kit (Thermo Scientific) according to manufacturer’s protocol. Primer sequences for the analysis of the transcript level of *CXXC4*, *DACT2*, and *HHIP* were designed using the Beacon Designer software and further BLAST searched to minimize non-specific binding. Each primer pair covered a region spanning two distinct exons. The sequences are listed in Table 4. Quantitative real-time PCR reactions were performed in a Chromo4 thermal cycler (Bio-Rad Laboratories, USA) using the HOT FIREPol Eva Green qPCR Mix (Solis BioDyne, Estonia). The amplification protocol was as follows: initial enzyme activation for 15 min at 95 °C followed by 40–45 cycles of 95 °C for 20 s, *X*°C for 20 s and 72 °C for 40 s, and the final elongation at 72 °C for 5 min, where *X* stands for the annealing temperature as described in Table 4. Subsequent melting curve analysis allowed the confirmation of the presence of a single product. Measurements were performed in duplicate, and mean values were normalized using *PBGD* (*porphobilinogen deaminase*) as the reference gene. Relative expression level was calculated with the ΔΔCt method using the formula: RE = 2 ^[Ct(ref) − Ct(test) TUMOR]^/2 ^[Ct(ref) − Ct(test) normal keratinocytes]^.

**Table 4:** The sequence of starters used in real-time PCR analysis

Gene	Starter sequence		Amplicon size	Annealing T
*CXXC4*	F	5′ CACCTCCTCCTCCTTCCAC	105 bp	56 °C
	R	5′ GCATTATCCTCCAACTGTTACAAC		
*DACT2*	F	5′ CAGGCTTTTACGAGATGAG	89 bp	56 °C
	R	5′ CAGCGAGGGAGAGATGTG		
*HHIP*	F	5′ TCCTATACCACCAACCAAGAAC	165 bp	59 °C
	R	5′ TTGTCCTCCCAGATGCTTTC		
*PBGD*	F	5′ TCAGATAGCATACAAGAGACC	111 bp	56 °C
	R	5′ TGGAATGTTACGAGCAGTG		

*F* forward primer, *R* reverse primer

### Statistical analysis

The association between clinicopathological variables and the occurrence of single gene methylation was analyzed using chi-square or Fisher’s exact tests. The association between clinicopathological variables and methylation index was analyzed using the Mann-Whitney *U* test or Kruskal-Wallis test. The analysis of the association of single gene methylation with overall survival time was performed using the *F*-Cox test. Multiple regression analysis allowed for the establishment of the association between methylation index and overall survival time. All the analyses were performed using the STATISTICA 10 software (StatSoft, Poland), and differences between groups were considered significant when *p* ≤ 0.05.

## Results

**Fig. 1:**
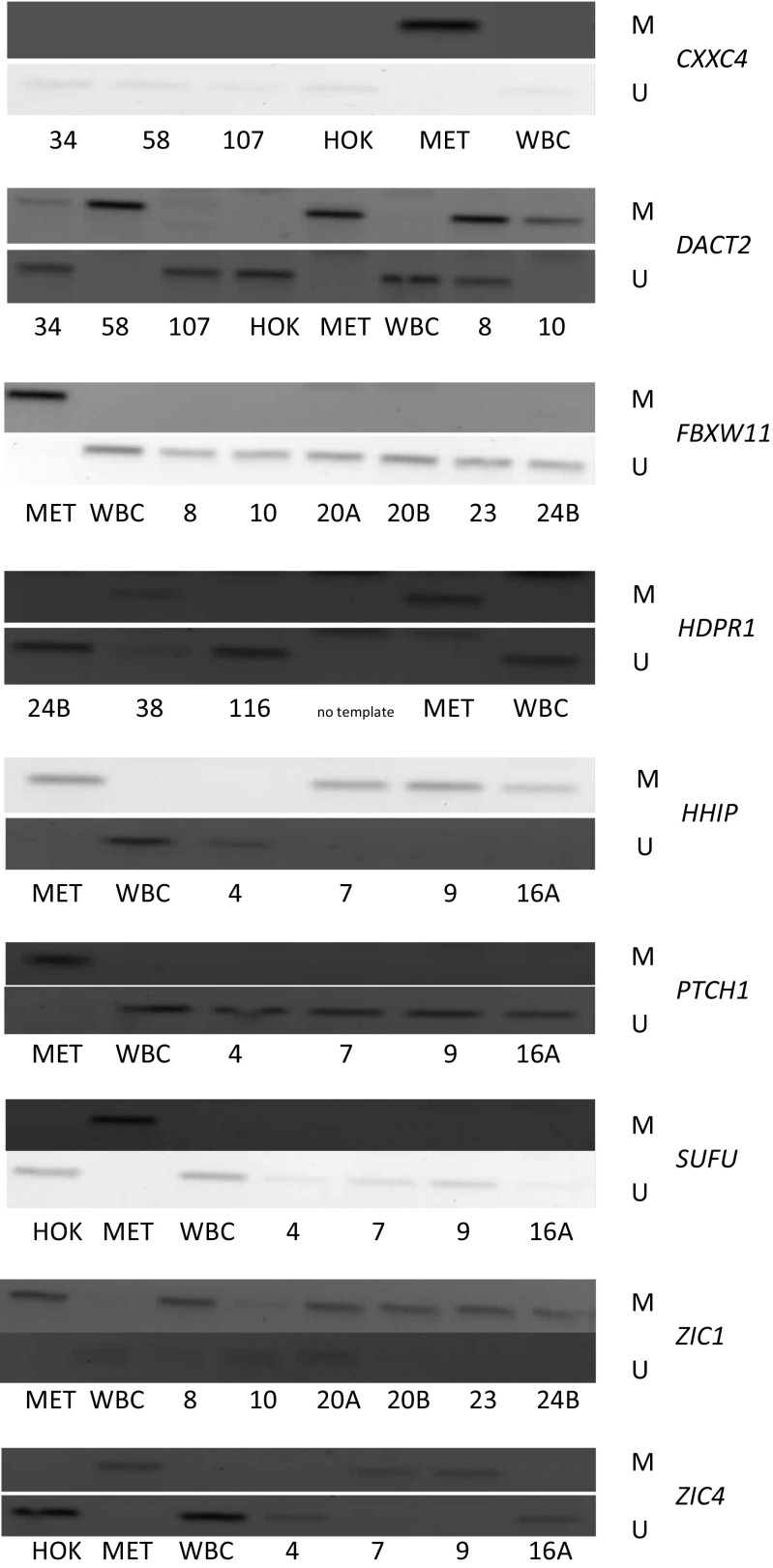
Representative electrophoregrams for the analysis of *CXXC4, DACT2, FBXW11, HDPR1, HHIP, PTCH1, SUFU, ZIC1*, and *ZIC4* methylation by methylation-specific PCR using starters specific for the methylated sequence (M) and starters specific for the unmethylated sequence (U) in head and neck squamous cell carcinoma cell lines and control DNA samples: *HOK* DNA isolated from healthy oral keratinocytes, *MET* completely methylated human DNA, *WBC* DNA derived from white blood cells of healthy blood donors

**Fig. 2:**
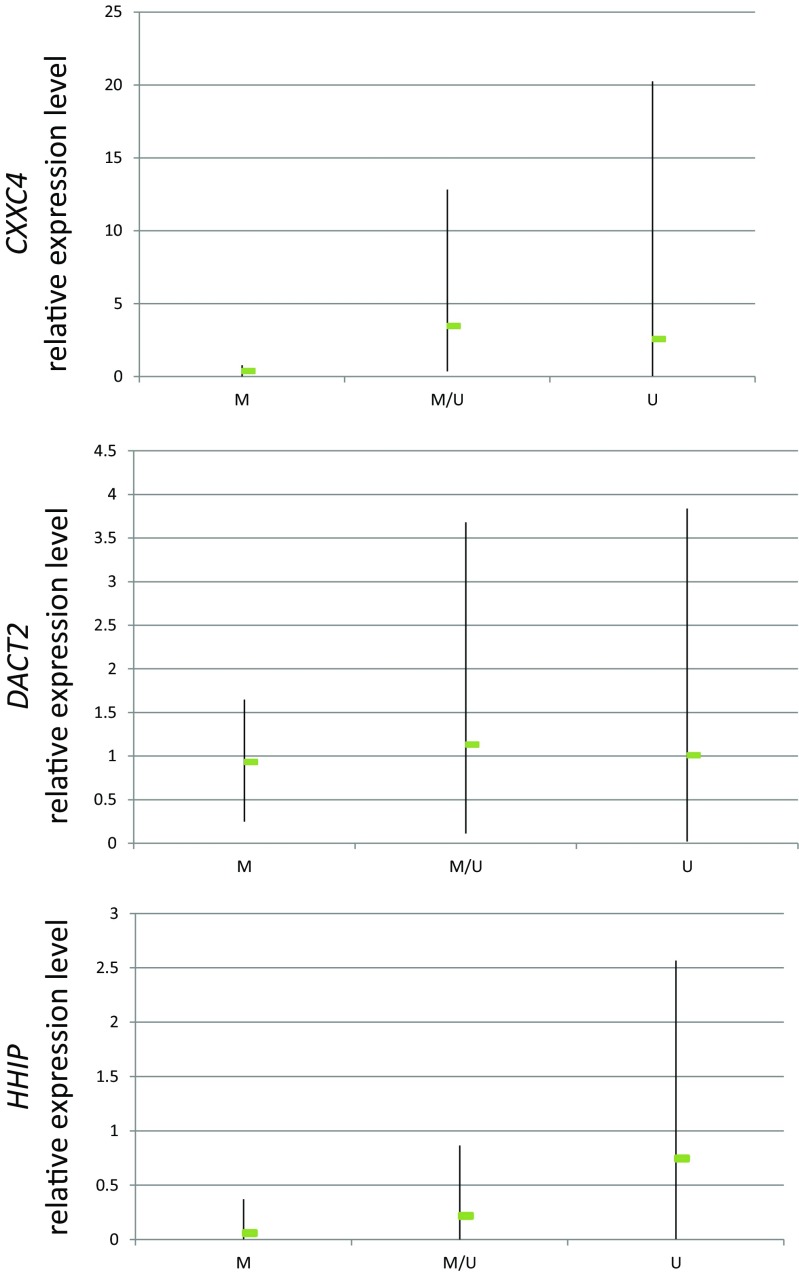
Relative expression levels of *CXXC4*, *DACT2*, and *HHIP* in HNSCC cell lines. The relative level of transcript was calculated as fold difference in relation to the expression in normal human keratinocytes (where expression was adopted as equal 1). The range of relative gene expression among cell lines (minimum and maximum values) and the mean level of relative expression (indicated in the graphs by the *arms* and the *green box*, respectively) in cell lines characterized by complete gene methylation (M), partial methylation (M/U), or lack of methylation (U) is shown

In the screening stage, the analysis of the occurrence of promoter methylation of the studied genes in DNA samples derived from head and neck squamous cell carcinoma cell lines allowed for the identification of those genes which undergo methylation in these tumors. No methylation was detected for *FBXW11*, *PTCH1*, or *SUFU* while all the other genes showed relatively low (*DACT2*) to high (*ZIC1*) frequency of methylation in the tested cell lines except *HDPR1*, which was only partly methylated in two cell lines (Table 5). Representative electrophoregrams are shown in Fig. 1. There was no statistically significant difference in the frequency of gene methylation between laryngeal and oral cavity and oropharyngeal carcinoma cell lines. Importantly, none of the genes was methylated in genomic DNA derived from normal oral keratinocytes or tracheal epithelial cells indicating that the methylation of *CXXC4*, *DACT2*, *HHIP*, *ZIC1*, and *ZIC4* does not appear in normal epithelial cells. We also wanted to assess whether the methylation of gene promoter regions under analysis correlated with their transcriptional activity. Therefore, we analyzed the expression of those genes which showed differential methylation status among the cell lines (Fig. 2). The expression of these genes in normal human oral keratinocytes (HOK) served as the reference in quantification. The quantitation of the transcript level of *CXXC4* and *HHIP* showed that full gene promoter methylation correlated with decreased level of expression when compared to expression in normal oral keratinocytes. The expression of these genes in cell lines showing only partial methylation or lack of methylation was not suppressed. This indicates that promoter methylation of these genes is associated with loss of their expression in HNSCC cells. Such an association was not observed in the case of *DACT2*, where the transcript level was similar irrespective of its methylation status indicating that the methylation of the analyzed promoter region is not the decisive factor in establishing transcription intensity.

**Table 5:** The frequency of gene promoter methylation in head and neck squamous cell carcinoma cell lines

Gene	Methylation status	Frequency (%)	Gene	Methylation status	Frequency (%)
*CXXC4*	M	17.2	*HHIP*	M	27.8
	M/U	22.8		M/U	30.5
	U	60		U	41.7
*DACT2*	M	11.4	*ZIC1*	M	88.9
	M/U	14.3		M/U	14.3
	U	74.3		U	2.8
*HDPR1*	M/U	5.7	*ZIC4*	M	22.2
	U	94.3		M/U	47.2
*FBXW11*	U	100		U	30.6
*PTCH1*	U	100	*SUFU*	U	100

*M* full gene methylation, *M/U* partial methylation of gene, *U* complete lack of gene methylation

**Fig. 3:**
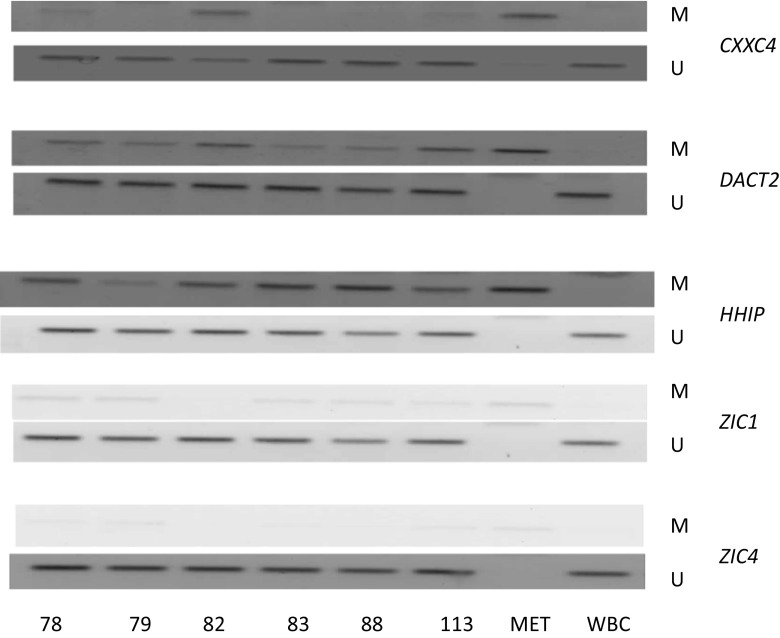
Representative electrophoregrams for the analysis of *CXXC4, DACT2, HHIP, ZIC1*, and *ZIC4* methylation by methylation-specific PCR using starters specific for the methylated sequence (M) and starters specific for the unmethylated sequence (U) in laryngeal tumor samples (patients no. 78, 79, 82, 83, 113) and control DNA samples: *MET* completely methylated human DNA, *WBC* DNA derived from white blood cells of healthy blood donors

Based on the findings that *CXXC4*, *DACT2*, *HHIP*, *ZIC1*, and *ZIC4* are methylated in HNSCC cell lines and that their methylation is not present in normal oral and tracheal epithelium, we further investigated the frequency of methylation of these genes in laryngeal and oral tumor samples and matched surgical margin samples of OSCC patients. Exemplary electrophoregrams are shown in Fig. 3, and the summary of the results is presented in Table 6. The rate of methylation of *ZIC1, ZIC4*, *HHIP*, and *DACT2* in tumors was very high, while methylation of *CXXC4* was low to moderate in OSCC and LSCC. Laryngeal tumors showed generally higher rates of gene methylation than oral cancers. Gene methylation was detected in all LSCC samples. The concurrent methylation of four genes was observed most frequently (43.7 %) followed by the observation of concurrent methylation of all five genes (23.3 %). The remaining cases showed the methylation of either three (20 %) or two (10 %) genes. Thus, the mean methylation index (MI—number of methylated genes divided by the number of all the tested genes) in LSCC samples was high—0.78. The occurrence of gene methylation was also highly prevalent among OSCC samples with only three cases showing the lack of methylation of any of the tested genes. Two OSCC cases showed the methylation of all five genes. The concurrent methylation of three genes was observed most frequently (26.2 %) followed by the concurrent methylation of two or four genes (23.8 %) or the methylation of a single gene (14.3 %). The mean methylation index in OSCC samples was 0.56. The frequency of methylation of the analyzed genes in OSCC surgical margin samples was lower than in the corresponding tumor samples what was reflected by a lower MI value of 0.4. The analysis of concordance (presence or lack of methylation in both paired samples) or discordance (presence of methylation in only one paired sample) of the results between paired tumor and surgical margin in OSCC patients is presented in Table 7. Most discordant results were associated with the lack of gene methylation in the resection margin.

**Table 6:** The frequency of gene promoter methylation in tumor and resection margin of HNSCC patients

	*CXXC4* (%)	*DACT2* (%)	*HHIP* (%)	*ZIC1* (%)	*ZIC4* (%)
LSCC	36.6	96.7	90	86.7	78.6
OSCC	10	53	62.5	100	73.2
OSCC margin	5.9	39.4	40	90	55.5

**Table 7:** The concordance of gene promoter methylation in tumor and resection margin of OSCC patients

	*CXXC4* (%)	*DACT2* (%)	*HHIP* (%)	*ZIC1* (%)	*ZIC4* (%)
T+ R+	0	31	23.7	89.5	51.4
T+ R*−*	5.9	17.2	36.8	10.5	22.8
T*−* R+	5.9	10.3	18.4	0	5.7
T*−* R*−*	88.2	41.4	21	0	20

*T* tumor, *R* resection margin, *+* presence of gene methylation, *−* lack of gene methylation

**Fig. 4:**
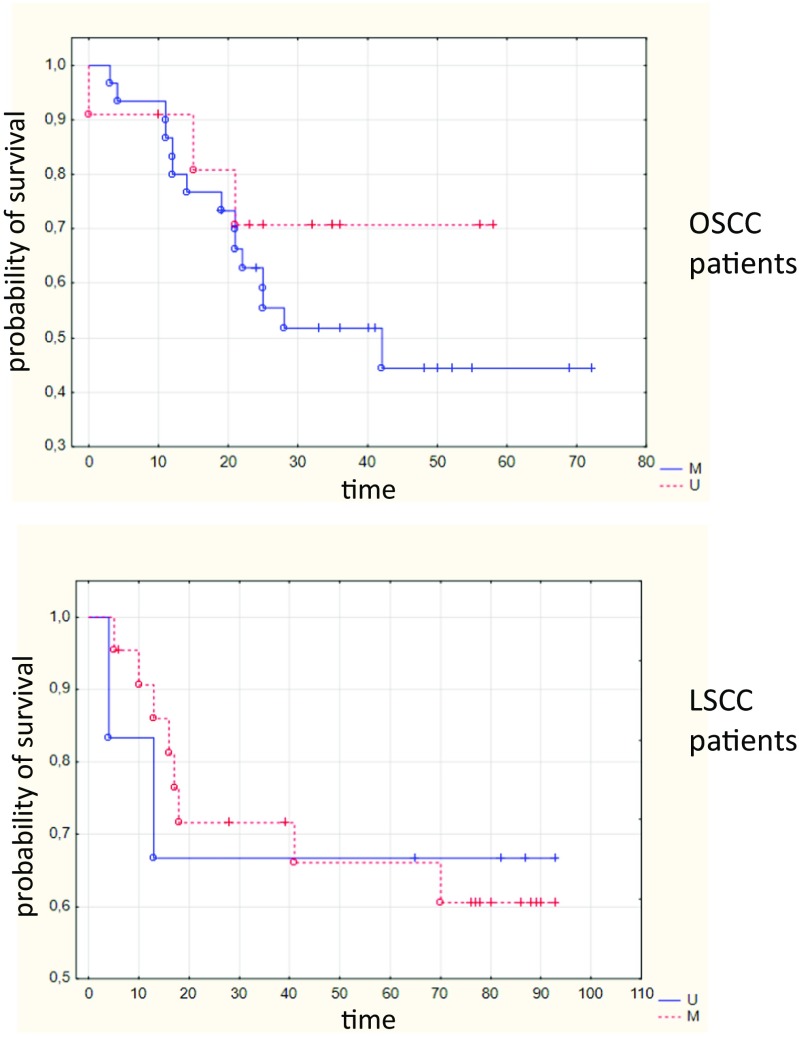
Overall survival estimated by *ZIC4* methylation detected in tumors of oral (OSCC) and laryngeal (LSCC) cancer patients. *M* patients showing *ZIC4* methylation; *U* patients showing lack of *ZIC4* methylation

The presence of gene methylation in tumor samples did not correlate with histological grade or tumor size. However, the presence of *ZIC4* methylation in oral tumors significantly correlated with lymph node invasion (*p* = 0.041). The methylation of *ZIC4* in tumors did not correlate with the overall survival of patients (Fig. 4) although there was a trend for an association between *ZIC4* methylation and shorter survival in OSCC patients (*p* = 0.09088). Moreover, the MI value in oral tumor samples moderately correlated with the overall time of survival in the subgroup of 18 patients who died of the disease (*r* = 0.46, *p* = 0.05). The presence of gene methylation in OSCC surgical margin did not show any statistically significant correlation with clinicopathological variables. However, the MI value was higher in the case of patients who developed second primary tumors. Although such an association was noted, it has to be considered dubious since there were only two patients in the studied group who developed a second primary tumor during follow-up observation.

## Discussion

Head and neck cancers are preventable tumors which develop mostly due to prolonged exposure to cigarette smoke and alcohol or HPV infection. In many cases, the tumor is diagnosed at a late stage when even aggressive treatment frequently fails to provide positive effects what results in low relapse-free and overall survival rates in patients with regional and distant head and neck carcinoma. Only recently, molecularly targeted therapies with EGFR (epithelial growth factor receptor) inhibitors have been applied with moderate outcomes in HNSCC indicating the need for the search of other therapeutic strategies. The increasing understanding of the molecular landscape in HNSCC points to several signaling pathways as drivers of the oncogenic transformation. Among those pathways, Wnt and Shh signaling seem especially interesting because of the possible association between their deregulation and disease progression. Infrequent mutations of members of these pathways and evidence of epigenetic silencing of their negative regulators stimulate the focus on the epigenetic mechanisms of modulation of the activity of these pathways in HNSCC.

Previous studies have shown that the enhanced transcriptional activity of β-catenin which is a marker of the activated canonical Wnt signaling is causally related to increased cell proliferation, migration, and epithelial-to-mesenchymal transition in HNSCC [9, 10]. Thus, the inhibition of β-catenin resulted in diminished cell growth and motility [19]. The hypermethylation of such negative regulators of Wnt pathway as *SFRP1–5*, *DKK1–3*, *WIF1*, *DACH1*, and *PPP2R2B* may significantly contribute to pathway activation and was frequently observed in HNSCC [15–18]. Moreover, both the increased expression of pathway components and the methylation of several antagonists correlated with worse prognosis. Based on these previous findings that the methylation of Wnt antagonists may have diagnostic significance, we decided to analyze the methylation of other genes (*CXXC4*, *DACT2*, *HDPR1*, *FBXW11*) which were not studied in this context in HNSCC. Wnt signaling is antagonized by DACT2 and HDPR1 proteins due to their ability to bind Disheveled and thus prevent the destabilization of the β-catenin inhibitory complex. The methylation of *DACT2* was previously reported in lung and also in gastric and thyroid cancers where it correlated with tumor differentiation and invasion [32, 36, 37]. On the other hand, *HDPR1* (*DACT1*) was downregulated by DNA methylation in breast or hepatocellular carcinoma [33, 38] and its downregulation was associated with poor prognosis in gastric and non-small cell lung cancers [39, 40]. Recently, it has been reported that both *DACT1* and *DACT2* are methylated in oral squamous cell carcinoma [41]. *CXXC4* encodes Idax protein which also antagonizes Wnt signaling by interacting with Disheveled. Its epigenetic downregulation led to the activation of Wnt signaling and was associated with poor outcome in gastric cancer patients [42]. *FBXW11* encodes one of the components of the complex responsible for the ubiquitylation of β-catenin leading to its subsequent degradation, and to our knowledge, the methylation of this gene has not been studied in cancer so far, but the promoter region of this gene contains a CpG island which possibly may become methylated. In our current study, the methylation of *HDPR1* was marginal in the HNSCC cell lines; thus, it was not further investigated in the group of HNSCC patients. Similarly, we did not detect the methylation of the promoter region of *FBXW11* in any of the tested HNSCC cell lines and thus did not investigate it further. On the other hand, we have observed that *DACT2* is frequently methylated in both the cells lines and head and neck carcinomas while *CXXC4* is methylated to a lesser extent. Moreover, their methylation is not observed in normal epithelial cells derived from healthy subjects. However, the methylation of *DACT2* or *CXXC4* did not significantly correlate with the clinicopathological features of the patients.

Few studies investigated the epigenetic regulation of Shh signaling in head and neck carcinomas; thus, we aimed to assess whether known negative regulators of this pathway (*HHIP*, *PTCH1*, *SUFU*, *ZIC1*, *ZIC4*) undergo methylation in HNSCC. HHIP inhibits Shh signaling by sequestering Hh ligands which then cannot activate the pathway, and its epigenetically mediated downregulation was observed in gastrointestinal, hepatocellular, and pancreatic tumors [43–46]. *PTCH1* blocks pathway activity in non-stimulated cells, and its loss due to methylation was observed in breast and gastric cancers and colorectal precancerous lesions [35, 46–48]. SUFU blocks the pathway by sequestering Gli transcription factors in the cytoplasm, and to our knowledge, its methylation has not been studied in cancer so far. *ZIC1* and *ZIC4* antagonize the transcriptional activity of Gli. Previous studies have shown that *ZIC1* is frequently methylated in colorectal, hepatocellular, and gastric cancers [49–51] while the methylation of *ZIC4* was observed in bladder cancer [52]. In our study, we have found lack of methylation of *PTCH1* or *SUFU* and frequent methylation of *HHIP*, *ZIC1*, and *ZIC4* in HNSCC. The observed lack of methylation of *PTCH1* is in contrast to a recent finding where restriction analysis of promoter methylation showed that this gene is methylated in many dysplastic lesions and nearly half of HNSCC cases [53]. Methodological differences may be one possible source of this discrepancy. Another study reported that *ZIC4* is methylated in a quarter of HNSCC cases [54]. To our knowledge, the methylation of *HHIP* has not been studied in HNSCC; however, it was reported that the expression of the encoded protein is lost in the majority of oral squamous cell carcinomas [55]. Thus, our observation that *HHIP* undergoes methylation may partly explain the mechanism of this loss in head and neck cancers. Importantly, *ZIC4* methylation in tumors significantly correlated with lymph node invasion implicating that it might be considered a prognostic biomarker. Assuming that *ZIC4* methylation leads to the upregulation of Gli1 signaling, it may be hypothesized that it would contribute to the induction of epithelial-to-mesenchymal transition what subsequently results in increased cell motility and invasion. Indeed, the enhancement in Shh-Gli1 signaling induced migration of pancreatic cancer cells [56, 57].

Epigenetic studies using DNA methylation-based markers frequently indicated that the use of a panel of genes has stronger diagnostic significance in comparison to single gene testing. In this regard, the panel consisting of *CXXC4*, *DACT2*, *HHIP*, *ZIC1*, and *ZIC4* is a promising starting point for the development of a prognostic epimarker panel based on the finding that the MI value in tumor samples moderately correlated with the overall time of survival in the subgroup of 18 OSCC patients who died of the disease. Interestingly, the observed MI value in the surgical margin was higher in the case of OSCC patients who developed second primary tumors. Although this should be interpreted with great caution due to a low number of patients who developed second primary tumor during follow-up, this would be in agreement with the field carcinogenesis model which assumes that a diversity of molecular changes is detectable in a broad field of affected mucosa what may predispose cells to further neoplastic transformation [58, 59]. In line with this model, epigenetic changes were observed not only in tumor sections but also in macroscopically normal surgical margin. The presence of epigenetic aberrations in surgical margin and distant mucosa in HNSCC is well documented [16, 60, 61]. These changes may constitute both part of the field of preneoplastic molecular alterations and be related to the presence of single neoplastic cells which are usually not detected under standard histopathological examination [61].

In summary, we report that *CXXC4*, *DACT2*, *HHIP*, *ZIC1*, and *ZIC4* are methylated in head and neck squamous cell carcinomas. These findings corroborate that the activation of Wnt signaling in HNSCC is related to epigenetic silencing of the negative regulators of this pathway. Moreover, the results of our study indicate that the same mechanism of activation may operate in the case of Shh signaling. In this regard, functional studies should follow to investigate whether epigenetic modulators may inhibit Wnt or Shh signaling due to the reactivation of expression of pathway antagonists. Additionally, we conclude that the methylation of *ZIC4* may be considered a new prognostic marker in HNSCC. Further investigations should determine the detailed diagnostic significance of methylation of *ZIC4*, *HHIP*, and *DACT2* in head and neck carcinomas.

## Abbreviations

HNSCC: Head and neck squamous cell carcinoma
LSCC: Laryngeal squamous cell carcinoma
OSCC: Oral squamous cell carcinoma

## References

[1] GLOBOCAN 2012 v1.0, Cancer incidence and mortality worldwide: IARC CancerBase No. 11 [Internet]. [database on the Internet]. International Agency for Research on Cancer. 2013. Available from: http://globocan.iarc.fr. Accessed: 19.09.2015

[2] Cancer in Poland in 2012 [database on the Internet]. Polish Registry of Cancer. 2014. Available from: http://onkologia.org.pl/wp-content/uploads/Rok2012.pdf. Accessed: 18.09.2015

[3] Leemans CR, Braakhuis BJ, Brakenhoff RH (2011). The molecular biology of head and neck cancer. Nature reviews. Cancer.

[4] Molinolo AA, Amornphimoltham P, Squarize CH, Castilho RM, Patel V, Gutkind JS (2009). Dysregulated molecular networks in head and neck carcinogenesis. Oral Oncol.

[5] Rothenberg SM, Ellisen LW (2012). The molecular pathogenesis of head and neck squamous cell carcinoma. J Clin Investig.

[6] Saito-Diaz K, Chen TW, Wang X (2013). The way Wnt works: components and mechanism. Growth Factors.

[7] Alvarado CG, Maruyama S, Cheng J (2011). Nuclear translocation of beta-catenin synchronized with loss of E-cadherin in oral epithelial dysplasia with a characteristic two-phase appearance. Histopathology.

[8] Ishida K, Ito S, Wada N (2007). Nuclear localization of beta-catenin involved in precancerous change in oral leukoplakia. Mol Cancer.

[9] Iwai S, Yonekawa A, Harada C, Hamada M, Katagiri W, Nakazawa M, Yura Y (2010). Involvement of the Wnt-β-catenin pathway in invasion and migration of oral squamous carcinoma cells. Int J Oncol.

[10] Lee SH, Koo BS, Kim JM (2014). Wnt/beta-catenin signalling maintains self-renewal and tumourigenicity of head and neck squamous cell carcinoma stem-like cells by activating Oct4. J Pathol.

[11] Ravindran G, Devaraj H (2012). Aberrant expression of beta-catenin and its association with DeltaNp63, Notch-1, and clinicopathological factors in oral squamous cell carcinoma. Clin Oral Investig.

[12] Cancer Genome Atlas Network (2015). Comprehensive genomic characterization of head and neck squamous cell carcinomas. Nature.

[13] Lea IA, Jackson MA, Li X, Bailey S, Peddada SD, Dunnick JK (2007). Genetic pathways and mutation profiles of human cancers: site- and exposure-specific patterns. Carcinogenesis.

[14] Pickering CR, Zhang J, Yoo SY (2013). Integrative genomic characterization of oral squamous cell carcinoma identifies frequent somatic drivers. Cancer Discov.

[15] Sogabe Y, Suzuki H, Toyota M (2008). Epigenetic inactivation of SFRP genes in oral squamous cell carcinoma. Int J Oncol.

[16] Paluszczak J, Hemmerling D, Kostrzewska-Poczekaj M (2014). Frequent hypermethylation of WNT pathway genes in laryngeal squamous cell carcinomas. J Oral Pathol Med.

[17] Paluszczak J, Sarbak J, Kostrzewska-Poczekaj M (2015). The negative regulators of Wnt pathway-DACH1, DKK1, and WIF1 are methylated in oral and oropharyngeal cancer and WIF1 methylation predicts shorter survival. Tumour Biol.

[18] Pannone G, Bufo P, Santoro A, Franco R (2010). WNT pathway in oral cancer: epigenetic inactivation of WNT-inhibitors. Oncol Rep.

[19] Duan Y, Fan M (2011). Lentivirus-mediated gene silencing of beta-catenin inhibits growth of human tongue cancer cells. J Oral Pathol Med.

[20] McMillan R, Matsui W (2012). Molecular pathways: the hedgehog signaling pathway in cancer. Clin Cancer Res.

[21] Cavicchioli Buim ME, Gurgel CA, Goncalves Ramos EA, Lourenco SV, Soares FA (2011). Activation of sonic hedgehog signaling in oral squamous cell carcinomas: a preliminary study. Hum Pathol.

[22] Dimitrova K, Stoehr M, Dehghani F (2013). Overexpression of the hedgehog signalling pathway in head and neck squamous cell carcinoma. Onkologie.

[23] Leovic D, Sabol M, Ozretic P (2012). Hh-Gli signaling pathway activity in oral and oropharyngeal squamous cell carcinoma. Head & neck..

[24] Schneider S, Thurnher D, Kloimstein P (2011). Expression of the sonic hedgehog pathway in squamous cell carcinoma of the skin and the mucosa of the head and neck. Head & neck..

[25] Wang YF, Chang CJ, Lin CP (2012). Expression of hedgehog signaling molecules as a prognostic indicator of oral squamous cell carcinoma. Head & neck.

[26] Yan M, Wang L, Zuo H (2011). HH/GLI signalling as a new therapeutic target for patients with oral squamous cell carcinoma. Oral Oncol.

[27] Mozet C, Stoehr M, Dimitrova K, Dietz A, Wichmann G (2013). Hedgehog targeting by cyclopamine suppresses head and neck squamous cell carcinoma and enhances chemotherapeutic effects. Anticancer Res.

[28] Fertig EJ, Markovic A, Danilova LV (2013). Preferential activation of the hedgehog pathway by epigenetic modulations in HPV negative HNSCC identified with meta-pathway analysis. PLoS One.

[29] Grenman R, Pekkola-Heino K, Joensuu H, Aitasalo K, Klemi P, Lakkala T (1992). UT-MUC-1, a new mucoepidermoid carcinoma cell line, and its radiosensitivity. Archives of otolaryngology—head & neck surgery.

[30] Herman JG, Graff JR, Myohanen S, Nelkin BD, Baylin SB (1996). Methylation-specific PCR: a novel PCR assay for methylation status of CpG islands. Proc Natl Acad Sci U S A.

[31] Li LC, Dahiya R (2002). MethPrimer: designing primers for methylation PCRs. Bioinformatics.

[32] Jia Y, Yang Y, Brock MV, Zhan Q, Herman JG, Guo M (2013). Epigenetic regulation of DACT2, a key component of the Wnt signalling pathway in human lung cancer. J Pathol.

[33] Yau TO, Chan CY, Chan KL (2005). HDPR1, a novel inhibitor of the WNT/beta-catenin signaling, is frequently downregulated in hepatocellular carcinoma: involvement of methylation-mediated gene silencing. Oncogene.

[34] Eichenmuller M, Gruner I, Hagl B (2009). Blocking the hedgehog pathway inhibits hepatoblastoma growth. Hepatology.

[35] Peng L, Hu J, Li S (2013). Aberrant methylation of the PTCH1 gene promoter region in aberrant crypt foci. Int j cancer.

[36] Yu Y, Yan W, Liu X (2014). DACT2 is frequently methylated in human gastric cancer and methylation of DACT2 activated Wnt signaling. Am J Cancer Res.

[37] Zhao Z, Herman JG, Brock MV (2014). Methylation of DACT2 promotes papillary thyroid cancer metastasis by activating Wnt signaling. PLoS One.

[38] Yin X, Xiang T, Li L (2013). DACT1, an antagonist to Wnt/beta-catenin signaling, suppresses tumor cell growth and is frequently silenced in breast cancer. Breast cancer res.

[39] Yang ZQ, Zhao Y, Liu Y (2010). Downregulation of HDPR1 is associated with poor prognosis and affects expression levels of p120-catenin and beta-catenin in nonsmall cell lung cancer. Mol Carcinog.

[40] Deng J, Liang H, Zhang R (2014). Methylated CpG site count of dapper homolog 1 (DACT1) promoter prediction the poor survival of gastric cancer. Am J Cancer Res.

[41] Schussel JL, Kalinke LP, Sassi LM (2015). Expression and epigenetic regulation of DACT1 and DACT2 in oral squamous cell carcinoma. Cancer Biomark.

[42] Lu H, Sun J, Wang F (2013). Enhancer of zeste homolog 2 activates wnt signaling through downregulating CXXC finger protein 4. Cell Death Dis.

[43] Taniguchi H, Yamamoto H, Akutsu N (2007). Transcriptional silencing of hedgehog-interacting protein by CpG hypermethylation and chromatic structure in human gastrointestinal cancer. J Pathol.

[44] Tada M, Kanai F, Tanaka Y (2008). Down-regulation of hedgehog-interacting protein through genetic and epigenetic alterations in human hepatocellular carcinoma. Clin Cancer Res.

[45] Martin ST, Sato N, Dhara S (2005). Aberrant methylation of the human hedgehog interacting protein (HHIP) gene in pancreatic neoplasms. Cancer Biol Ther.

[46] Song Y, Tian Y, Zuo Y, JC T, Feng YF, Qu CJ (2013). Altered expression of PTCH and HHIP in gastric cancer through their gene promoter methylation: novel targets for gastric cancer. Mol Med Rep.

[47] Zuo Y, Song Y (2013). Detection and analysis of the methylation status of PTCH1 gene involved in the hedgehog signaling pathway in a human gastric cancer cell line. Exp Ther Med.

[48] Wolf I, Bose S, Desmond JC (2007). Unmasking of epigenetically silenced genes reveals DNA promoter methylation and reduced expression of PTCH in breast cancer. Breast Cancer Res Treat.

[49] Gan L, Chen S, Zhong J (2011). ZIC1 is downregulated through promoter hypermethylation, and functions as a tumor suppressor gene in colorectal cancer. PLoS One.

[50] Wang YY, Jiang JX, Ma H (2014). Role of ZIC1 methylation in hepatocellular carcinoma and its clinical significance. Tumour Biol.

[51] Wang LJ, Jin HC, Wang X (2009). ZIC1 is downregulated through promoter hypermethylation in gastric cancer. Biochem Biophys Res Commun.

[52] Kandimalla R, van Tilborg AA, Kompier LC (2012). Genome-wide analysis of CpG island methylation in bladder cancer identified TBX2, TBX3, GATA2, and ZIC4 as pTa-specific prognostic markers. Eur Urol.

[53] Ghosh A, Ghosh S, Maiti GP (2012). Association of FANCC and PTCH1 with the development of early dysplastic lesions of the head and neck. Ann Surg Oncol.

[54] Guerrero-Preston R, Michailidi C, Marchionni L, et al. (2014) Key tumor suppressor genes inactivated by “greater promoter” methylation and somatic mutations in head and neck cancer. Epigenetics 9(7). doi:10.4161/epi.29025 10.4161/epi.29025 PMC4143405 24786473

[55] Snijders AM, Schmidt BL, Fridlyand J (2005). Rare amplicons implicate frequent deregulation of cell fate specification pathways in oral squamous cell carcinoma. Oncogene.

[56] Xu X, Zhou Y, Xie C (2012). Genome-wide screening reveals an EMT molecular network mediated by sonic hedgehog-Gli1 signaling in pancreatic cancer cells. PLoS One.

[57] Xu X, Su B, Xie C (2014). Sonic hedgehog-Gli1 signaling pathway regulates the epithelial mesenchymal transition (EMT) by mediating a new target gene, S100 A4, in pancreatic cancer cells. PLoS One.

[58] Dotto GP (2014). Multifocal epithelial tumors and field cancerization: stroma as a primary determinant. J Clin Invest.

[59] Mohan M, Jagannathan N (2014). Oral field cancerization: an update on current concepts. Oncol Rev.

[60] Paluszczak J, Misiak P, Wierzbicka M, Wozniak A, Baer-Dubowska W (2011). Frequent hypermethylation of DAPK, RARbeta, MGMT, RASSF1A and FHIT in laryngeal squamous cell carcinomas and adjacent normal mucosa. Oral Oncol.

[61] Mielcarek-Kuchta D, Paluszczak J, Seget M (2014). Prognostic factors in oral and oropharyngeal cancer based on ultrastructural analysis and DNA methylation of the tumor and surgical margin. Tumour biol.

